# Preparation and Performance of Thermochromic and Self-Repairing Dual Function Paint Film with Lac Resin Microcapsules and Fluorane Microcapsules

**DOI:** 10.3390/polym13183109

**Published:** 2021-09-15

**Authors:** Xiaoxing Yan, Wenting Zhao, Lin Wang

**Affiliations:** 1 Co-Innovation Center of Efficient Processing and Utilization of Forest Resources, Nanjing Forestry University, Nanjing 210037, China; 2 College of Furnishings and Industrial Design, Nanjing Forestry University, Nanjing 210037, China; zhaowenting@njfu.edu.cn (W.Z.); wanglin@njfu.edu.cn (L.W.)

**Keywords:** lac resin microcapsule, fluorane microcapsule, paint film, discoloration, self-repair

## Abstract

Microcapsules with lac resin as the core material and urea-formaldehyde resin as the wall material were prepared by in situ polymerization, and then the lac resin microcapsules and fluorane microcapsules were added into a water-based primer or topcoat, respectively, to prepare water-based coatings with dual functions of thermochromic and self-repair. The effects of different methods of adding microcapsules on the optical properties, mechanical properties, self-repairing properties, and the aging resistance of water-based paint film were investigated, so as to prepare water-based paint film with the best discoloration and self-repairing functions. The results showed that the paint film with 10.0% fluorane microcapsules in the topcoat and 5.0% lac resin microcapsules in the primer had better comprehensive properties, and the paint film changed from yellow to colorless at 32 °C, with a color difference of 68.9, hardness of 3H, adhesion grade of 0, impact resistance of 13.0 kg∙cm, and elongation at break of 20.0%. The resistance of the paint film to NaCl, ethanol, and detergent was grade 2, with slight discontinuous marks, and the resistance to red ink was grade 3, with slight marks. The lac resin microcapsules have good aging resistance, which can enhance the aging resistance of the paint film with fluorane microcapsules. The gap width of the paint film was repaired by 2.1 µm, the self-repairing rate was 12.3%, and the paint film with lac resin microcapsules had a better crack inhibition effect. The results have provided a reference for multifunctional wood coatings.

## 1. Introduction

As a natural polymer composite material, wood material has a unique vein, color, and texture and is the main material for furniture [1,2,3]. Coatings on the furniture surface can effectively improve the dry shrinkage and wet swelling of wood and play a further role in aesthetics and protection [4,5,6,7]. However, in daily life, due to the destruction of environmental factors and human factors, the paint film on the surface of furniture will produce microcracks that are hard to see with the naked eye, and the moisture and oxygen in the air will enter the wood, which will destroy the dimensional stability of wood and shorten the service life of wood in the long run. A self-repairing microcapsule is a kind of foreign aid repairing material. Once the paint film is damaged on the outside, the microcapsules will break, and the repairing agent will flow out, thus repairing the cracks.

Lac is a natural product which is nontoxic and environmentally friendly, has a good film-forming property, is fast drying and curing, and has unique solubility and excellent thermal properties [8]. Lac coatings made of lac are also widely used in the field of wood furniture [9,10]. Hamad et al. [11] reported a new preparation method of shellac-cell composite microcapsules, which can realize the programmed release of cells in a narrow pH range. It can be applied to the protection and delivery formula of probiotics and other cell cultures and releases encapsulated cells through programming and triggering in cell implants. Long et al. [12] used calcium shellac (CS) as a matrix to encapsulate polymerized melamine formaldehyde microcapsules or CaCO_3_ nanoparticle-stabilized microcapsules to prepare composite microcapsules with enhanced mechanical stability and reduced active ingredient leakage. The mechanical stability of composite microcapsules was improved, and the leakage of active ingredients was reduced by an order of magnitude. Based on the hydrophobicity and acid resistance of shellac, Huang et al. [13] designed shellac microcapsules with lactic acid bacteria ReuteriTMW1.656 to provide good moisture resistance and controlled release in the digestive tract. The results showed that the addition of shellac helped to improve the survival rate of probiotics after freeze-drying and simulated digestion, heating, and storage at room temperature, and the addition of whey protein isolate (WPI) had a synergistic effect. From the above studies, it can be seen that lac is usually used as the wall material of microcapsules in the fields of medicine and food. There were few studies using lac as the core material for self-repairing microcapsules.

Thermochromic wood is coated with thermochromic coatings on the surface, which makes the color of wood change with the temperature and meets people’s pursuit of intelligent and diversified wood products. The thermochromic microcapsule is a kind of material with an intelligent response, which can change its color with the change of ambient temperature [14]. That is, when the microcapsules are raised to a specific temperature, they can change from one color to another, and then they can change to the original color when the temperature drops [15]. Park et al. [16] prepared two kinds of phase change materials containing polyurea microcapsules in the core to realize the gradient color change of microcapsules, and the color changed gradually from dark to light blue, showing potential application prospects in color convertible intelligent windows. Raesi et al. [17] encapsulated the solution of spironaphthoxazine in oleic acid by the solvent evaporation method and encapsulated the microcapsules in a transparent polymer coating and non-woven fabric. The anti-isomerization rate, photo-induced patterning, and thermal regulation characteristics of spironaphthoxazine microcapsules were systematically studied. Under the irradiation of ultraviolet-visible light below the melting point of oleic acid, the microcapsules showed reversible color change, and compared with microcapsules without oleic acid, the color change rate decreased by 85%. Ma et al. [18] combined thermochromic materials with optimized disperse cationic dyes and designed a new type of thermochromic microcapsule to expand the color range of thermochromic materials. Thermochromic microcapsules have mainly focused on the reversible direction at low temperatures and have mainly been used in the textile and machinery fields, but there have been a few application cases in wood furniture.

Fluorane dyes in fluorane microcapsules have the advantages of low discoloration temperature, high sensitivity, and stable discoloration performance, which can realize the thermochromism between yellow and colorless and meet the requirements of wood discoloration. Lac resin dissolved in ethanol can be used as a core material of microcapsules to repair cracks at room temperature [19,20,21]. With the seasonal change of indoor temperature, the surface of wood products will automatically change color. The coatings can realize the changeable effect and style of indoor decoration. At the same time, if there are micro-cracks in the use of wood products and wood furniture, they can be repaired automatically to prolong the service life of wood products.

In this paper, microcapsules with urea-formaldehyde resin as the wall material and lac resin as the core material were prepared by in situ polymerization. In order to make the paint film have better color-changing performance and without reducing the original performance of the paint film, three factors, “fluorane microcapsule content”, “lac resin microcapsule content”, and “microcapsule adding method”, were selected to design three-factor and two-level orthogonal experiments to prepare paint films. The application of these two microcapsules in water-based wood coatings can expand the application range of microcapsules and provide reference for multifunctional coatings.

## 2. Materials and Methods

### 2.1. Experimental Materials

The experimental materials are shown in Table 1. Basswood had the specification of 100 mm × 50 mm × 5 mm. After pre-sanding, the surface was smooth, and the color of wood was uniform. Thermochromic microcapsules (fluorane microcapsules) had a color change temperature of 31 °C. The main components were melamine formaldehyde resin and 1,2-benzo-6-diethylamino fluorane. The main components of Dulux water-based primer and Dulux water-based topcoat were water-based acrylic resin, and the solid content was about 30.0%.

### 2.2. Experimental Method

#### 2.2.1. Preparation of Lac Resin Microcapsules

The 30.0 g of urea and 40.5 g of formaldehyde were stirred to be fully dissolved, and triethanolamine was added to adjust the pH to 9. The wall material prepolymer solution can be obtained by stirring in a magnetic stirrer at 68 °C for 70 min. The 2.6 g of sodium dodecyl benzene sulfonate and 261.0 mL of distilled water were stirred to complete dissolution. The 33.8 g of lac resin and 168.8 mL of ethanol were stirred until fully dissolved. The sodium dodecylbenzene sulfonate solution was added into the lac solution and stirred at 1200 rpm for 35 min to obtain a uniform emulsion core solution. At the rotating speed of 400 rpm, the wall material solution was added into the core material solution, citric acid was added to adjust the pH to 2, and then the solution was reacted for 2.5 h. After the reaction, the solution was left for 7 d and filtered by suction. The rest of the products were dried in an oven to obtain lac resin microcapsules.

#### 2.2.2. Preparation of Thermochromic and Self-Repairing Bifunctional Paint Film

**Figure 1:**
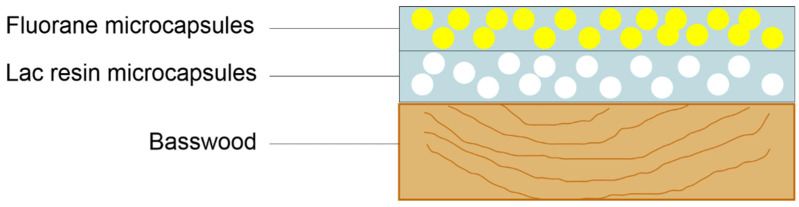
Schematic diagram of paint film.

Three-factor and two-level orthogonal experiments were designed with “fluorane microcapsule content”, “lac resin microcapsule content”, and “microcapsule adding method”, as shown in Table 2. Through pre-experiment, it can be seen that when 15.0% fluorane microcapsules were added to water-based coatings, the thermochromic performance of paint film on Basswood surface was better, so the horizontal range of “fluorane microcapsule content” was 10.0–20.0% to determine the best value. At the same time, according to the previous research, it was determined that the comprehensive performance of the paint film was better when the microcapsule content was 10.0% [22], so the horizontal range of “lac resin microcapsule content” in this experiment was 5.0–15.0%. The ingredients of water-based coatings with two kinds of microcapsules are shown in Table 3. Samples 1–4 were orthogonal experimental samples. Samples 1 and 5 were independently optimized experimental samples based on orthogonal experiments. Samples 6–8 were blank control samples. Schematic diagram of paint film is shown in Figure 1. Microcapsules and water-based coatings with corresponding weight were weighed, stirred evenly, coated on Basswood substrate, dried at room temperature for 30 min, polished with sandpaper to wipe off floating powder, and then coated again according to the above steps. The dry film thickness was about 60.0 µm.

### 2.3. Testing and Characterization

The test instruments for this experiment were shown in Table 4. Heating plate and hand-held thermometer were used to heat and measure the temperature of paint film samples. The SEGT-J portable colorimeter was used to measure the color difference change of the paint film at 16–40 °C [23]. *L* represents the lightness, *a* represents the red-green value, and *b* represents the yellow-blue value. The larger the *L* value, the brighter the paint film; the larger the *a* value, the redder the paint film; the larger the *b* value, the yellower the paint film. The chromaticity value of the sample at 16 °C was the reference basic point, and the chromaticity values at different temperatures during heating were recorded. According to color difference equation, the color difference (Δ*Ε*) of the sample at different temperatures was calculated:

(1) $$\Delta E=\sqrt{{\left(L_{1}-L_{2}\right)}^{2}+{\left(a_{1}-a_{2}\right)}^{2}+{\left(b_{1}-b_{2}\right)}^{2}}$$

The hardness of the paint film was measured by QHQ portable pencil hardness tester. First, 6H-6B pencils were prepared, among which 6H pencil was the hardest and 6B pencil was the softest. The wooden pole of the pencil was cut off with a pencil sharpener, so that the lead core was cylindrical and exposed about 3.0 mm. Then, the lead was ground by sandpaper until the tip of the pencil was flat and the edge was sharp, and the pencil was fixed on the pencil clamp. The paint film of the sample was facing upwards, placed horizontally, and fixed. The 1.0 ± 0.05 kg of weight was placed on the platform so that the tip of the pencil core contacted the paint film surface, and the load of the weight was added to the tip. When the hand wheel was shaken at a constant speed, the test sample moved about 3.0 mm to make the refill scratch the paint film surface, and the moving speed was 0.5 mm/s. Five passes were scratched. The pencil with two or more scratches on the coating film was selected, and the hardness label of the next pencil was recorded [24].

According to GB/T 4893.4-2013 [25], the adhesion of the paint film was measured by the HGQ film scratch tester. The paint film was cut with grid pattern by a single-edged grid cutter with a spacing of 2.0 mm, so that it just penetrated the substrate. The adhesive tape with a width of 25.0 mm and an adhesive force of 10 N was attached to the center of the grid, and the adhesive tape was torn off. The adhesive force level was judged according to the area of the paint film falling off the substrate in the grid area. The adhesion grade was 0~5, with 0 being the best and 5 being the worst.

According to GB/T 1732-1993 [26], QCJ-50 coating impact tester was selected to test the impact resistance of paint film. The test piece was placed on the horizontal base, the impact block was lifted to a certain height, and then the impact test piece was dropped, and the damage degree of the test piece was checked to determine the impact resistance.

The elongation at break of the film was measured by HY-0580 elongation at break tester. The paint film was fixed with clamps, so that the paint film lay on the vertical plane of the upper and lower clamps, the distance *L_1_* between the lower and upper clamps was tested, the machine was started, and the machine was stopped when the paint film broke. At this time, the clamp distance *L_2_* was read, and the elongation at break (*e*) was calculated according to Formula (2):

(2) $$e=\frac{L_{2}-L_{1}}{L_{1}}\times 100\%$$

According to GB/T 4893.1-2005 [27], NaCl solution, ethanol, detergent, and red ink were used to determine the liquid resistance of paint film. The filter paper was soaked in solution, placed on the paint film sample, and covered with a glass cover. After 24 h, the filter paper was removed, and the damage of the paint film surface was observed to determine the liquid resistance grade. The microstructure was observed with Axio Scope A1 optical microscope and KYKY-EM6900 scanning electron microscope. The chemical constituents were analyzed by XLPE Fourier transform infrared spectrometer.

The aging resistance test of the paint film was carried out in an oven at 120 °C and 160 °C and the ultraviolet weather resistance test oven. The color difference of the paint film was tested every 8 h in the oven for 40 h. The color difference of the paint film was tested every 40 h in the UV weathering test box for 200 h. The color difference and surface morphology of the paint film before and after aging were observed and compared. The self-repairing rate of paint film was characterized by scratch test. The blade was used to draw a crack artificially on the surface of paint film. The width of crack was observed under microscope, which was expressed as *A_original_*. After 7 d, the crack width was observed again, and the crack width was expressed as *A_healed_*. The self-repairing rate of the crack was calculated according to Formula (3):

(3) $$\sigma =\frac{A_{original}-A_{healed}}{A_{original}}\times 100\%$$

All the experiments were repeated 4 times, and the error was less than 5.0%.

## 3. Results

### 3.1. Morphology and Properties of Lac Resin Microcapsules

**Figure 2:**
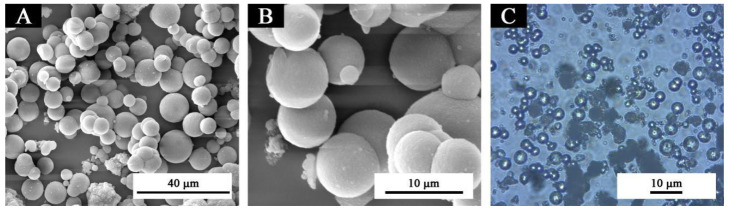
Morphology of lac microcapsules: SEM of (**A**) low magnification, (**B**) high magnification, and (**C**) OM of lac resin microcapsules.

**Figure 3:**
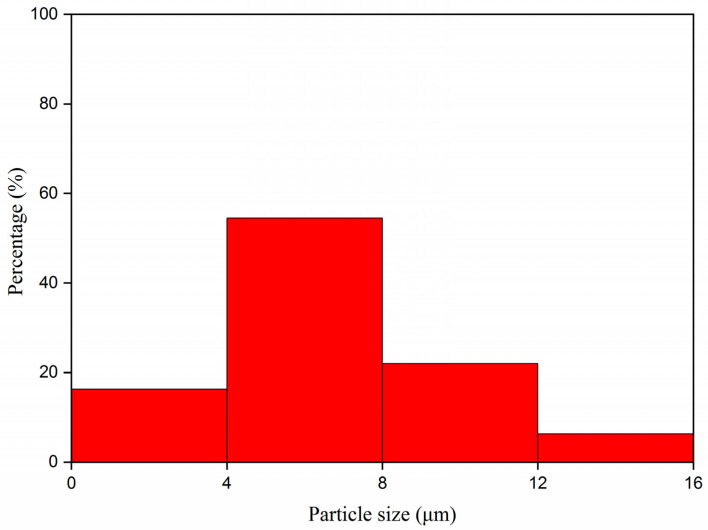
Particle size distribution of lac resin microcapsules.

SEM and OM diagrams of lac resin microcapsules prepared by in situ polymerization are shown in Figure 2. It is shown that the microcapsules were round and spherical (Figure 2A,B). Figure 2C shows the microcapsules observed under the optical microscope. It can be seen that the brighter part in the middle was the core material, and the darker part outside was the wall material [28]. The microcapsule was nearly round. The particle size distribution of microcapsules is shown in Figure 3. About 60% of the microcapsules are in the range of 4–8 µm, and the particle size was relatively uniform.

**Figure 4:**
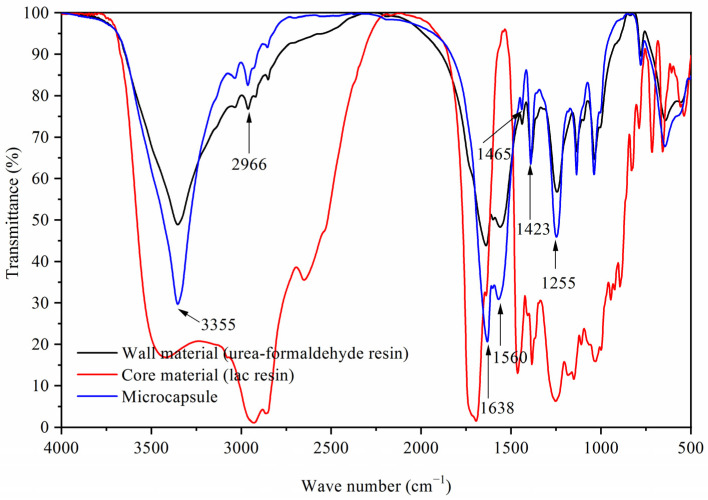
FTIR of urea formaldehyde resin, lac resin, and lac resin microcapsule.

Figure 4 shows the infrared spectra of the lac resin, urea-formaldehyde resin, and lac resin microcapsules. The absorption peaks at 3355 cm^−1^ and 2966 cm^−1^ were due to the tensile vibration of N-H and C-H, and the absorption peaks at 1638 cm^−1^ and 1560 cm^−1^ were due to the tensile vibration of C=O and C-N. These four characteristic peaks proved the existence of urea formaldehyde resin [29]. The absorption peaks at 1465 cm^−1^, 1423 cm^−1^, and 1255 cm^−1^ were the absorption peaks of lac resin [30,31]. Infrared spectra showed that the microcapsules with lac resin as the core material and urea-formaldehyde resin as the wall material were successfully prepared.

### 3.2. Orthogonal Experimental Analysis

**Figure 5:**
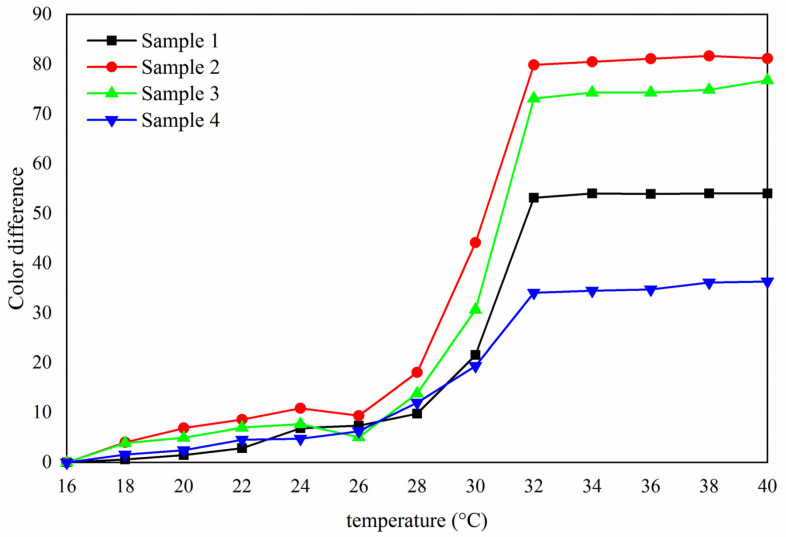
Variation trend of color difference with temperature (16–40 °C).

It can be seen from Figure 5 that with the increase of temperature, the color difference of samples 1–4 gradually increased, and the color difference of orthogonal samples 2 and 3 was much larger than that samples of 1 and 4. On the whole, when the temperature was in the range of 16–30 °C, the color difference of the paint film did not change much. When the temperature rose from 30 °C to 32 °C, the color difference amplitude of the paint film increased and tended to a maximum at 32 °C, which indicated that the thermochromic phenomenon occured at 32 °C.

In order to make the paint film have the best thermochromic performance, the color difference of 16–32 °C was brought into the orthogonal analysis. The orthogonal experiment and extreme difference results (Table 5) showed that “the way of adding microcapsules” was the primary factor that affected the color difference of the paint film. According to the mean value 1 and mean value 2, it can be further known that the color difference of the paint film was greater when the content of fluorane microcapsules was 10.0% and that of lac resin microcapsules was 5.0%. Therefore, in the next optimization experiment, the content of fluorane microcapsules was 10.0% and that of lac resin microcapsules was 5.0%, and the effects of fluorane microcapsules on various properties such as discoloration and self-repair of the paint film were studied by changing the adding methods of fluorane microcapsules.

### 3.3. Single Factor Experimental Results and Analysis of “Adding Method of Fluorane Microcapsules”

**Figure 6:**
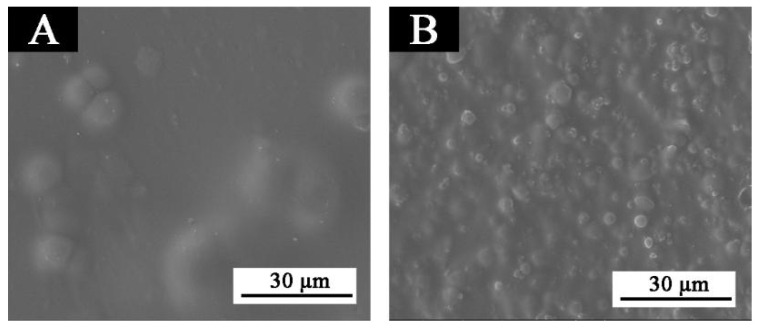
SEM of paint film with different adding methods: (**A**) sample 1, (**B**) sample 5.

Figure 6A shows the paint film with primer added with 10.0% fluorane microcapsules and topcoat added with 5.0% lac resin microcapsules, and Figure 6B shows the paint film with topcoat added with 10.0% fluorane microcapsules and primer added with 5.0% lac resin microcapsules. It can be seen that the way of adding microcapsules had a certain influence on the paint film. The results showed that the particles in the paint film with 10.0% fluorane microcapsules were very obvious, while the paint film with 5.0% lac resin microcapsules was smooth.

**Figure 7:**
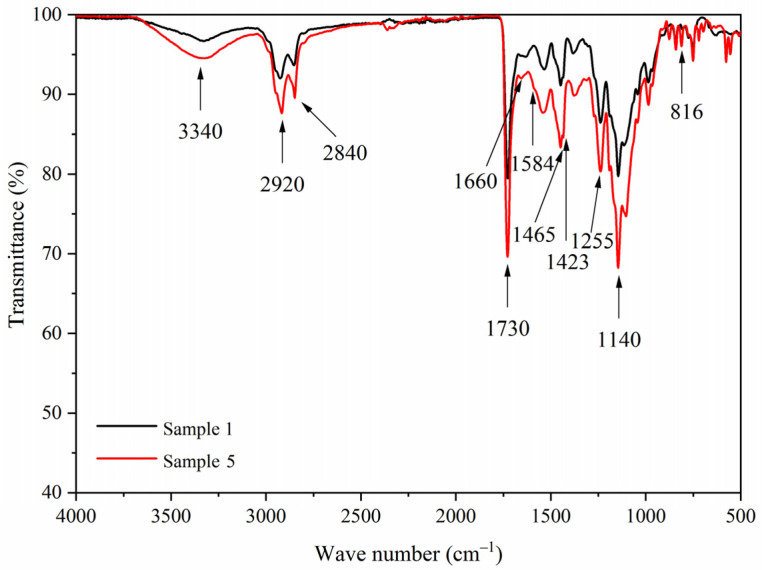
FTIR of paint film with different adding methods.

Figure 7 shows infrared spectra of samples 1 and 5 paint films. The 1660 cm^−1^ was the stretching vibration of C=O in UF resin. The 1730 cm^−1^ with strong carbonyl absorption represents the characteristic peak of C=O of 1,2-benzo-6-diethylamino fluorane, which was one of the core materials of fluorane microcapsules. At 1465 cm^−1^ and 1423 cm^−1^, there were COO— antisymmetric and symmetric stretching vibrations of carbonyl anion of carboxylic acid. At 1255 cm^−1^, there are C=O-C stretching vibrations of ester molecule. The 1465 cm^−1^, 1423 cm^−1^, and 1255 cm^−1^ were the characteristic peaks of lac resin. The characteristic peaks of the fluorane microcapsule and lac resin microcapsule were found in samples 1 and 5 infrared spectra. Additionally, no peak disappeared or appeared, indicating that there was no difference in paint film composition with different addition methods.

**Figure 8:**
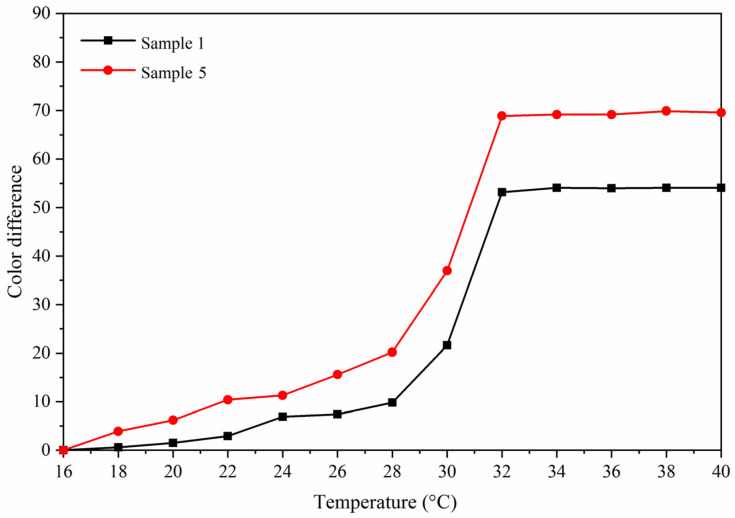
The graph of color difference with temperature increase.

**Figure 9:**
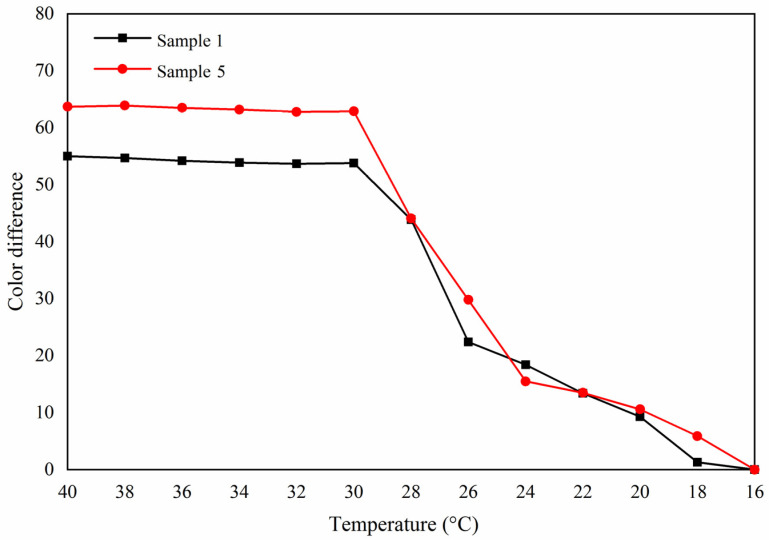
The graph of color difference with temperature decrease.

Figure 8 is a trend diagram of color difference with temperature. The color difference of samples 1 and 5 paint films gradually increased with the increase of temperature. When the temperature reached 32 °C, the color difference of the paint film was the largest, and the paint film completely changed color. At this time, the color difference of sample 1 reached 53.2 and of sample 5 reached 68.9. According to the chromatic aberration trend during heating and cooling (Figure 8 and Figure 9), the results showed that the color-changing temperature range could not be changed by adding methods, and the prepared paint film could still realize reversible thermochromism. The sample 5 with 10.0% fluorane microcapsules in the topcoat and 5.0% lac resin microcapsules in the primer had an obvious color change.

Table 6 showed the influence of the ways of adding fluorane microcapsules on the impact resistance, hardness, elongation at break, and adhesion of the paint film. The adhesion of samples 1 and 5 was grade 0, the hardness was 3H, and the impact resistance was basically the same. The elongation at break of sample 1 was 10.0%, which was lower than 20.0% of sample 5. This may be because when the paint film was stretched, the lac resin microcapsules in the topcoat were not yet repaired, and the paint film with lac resin microcapsules added in the primer had a buffer effect, which led to higher flexibility of the paint film with lac resin microcapsules added in the topcoat [32].

The liquid resistance tests of samples 1 and 5 against NaCl, detergent, ethanol, and red ink were carried out, and the experimental results are shown in Table 7. The liquid resistance of sample 1 to ethanol was grade 1, the paint film was not damaged, and the liquid resistance to NaCl and detergent was grade 2, with slight discontinuous marks on the paint film surface. The resistance of sample 5 to NaCl, detergent, and ethanol was grade 2, and there were slight discontinuous marks on the paint film. The two kinds of paint film have poor liquid resistance to red ink, which was grade 3, and there were slight marks on the paint film. The results showed that red ink had a greater influence on the paint film. This was because the film was originally yellow at room temperature. After the red ink test, the color of the paint film changed from yellow to red by capillary action, so the color difference became larger, leaving a more obvious impression. According to the performance analysis of the single factor experiment of “adding method of fluorane microcapsules”, it can be seen that the comprehensive performance of sample 5 was the best.

### 3.4. Analysis of Aging Resistance

**Figure 10:**
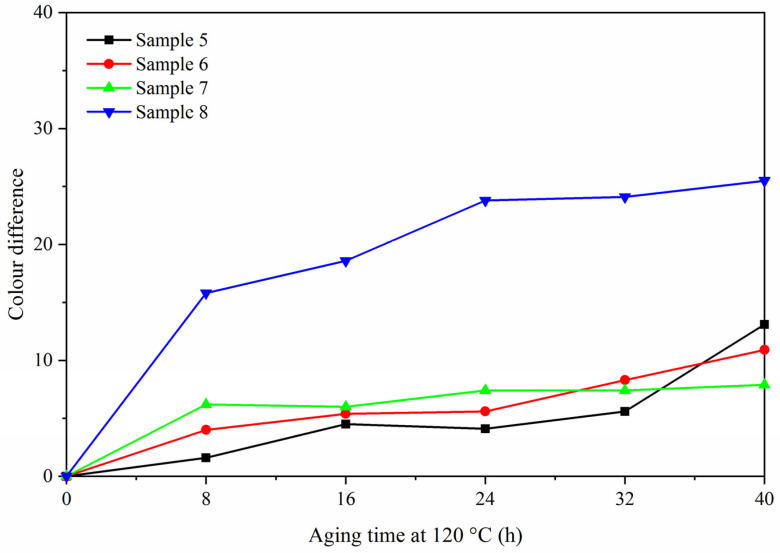
Variation trend of color difference with aging time at 120 °C.

**Figure 11:**
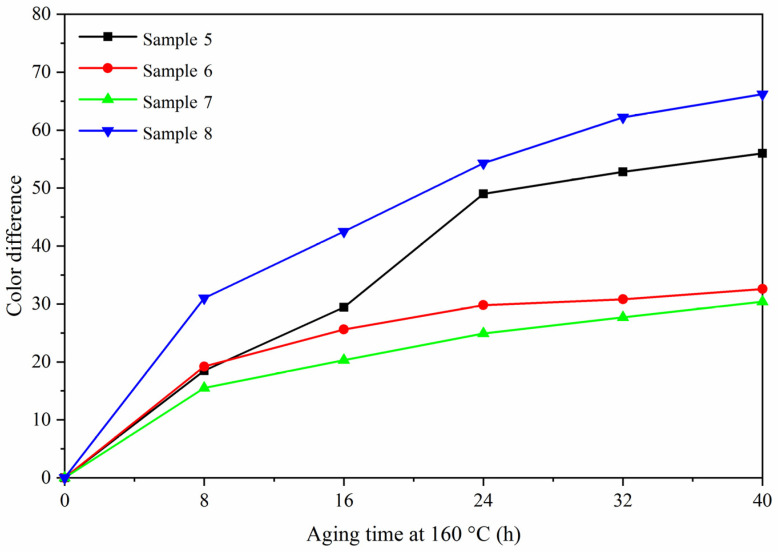
Variation trend of color difference with aging time at 160 °C.

**Figure 12:**
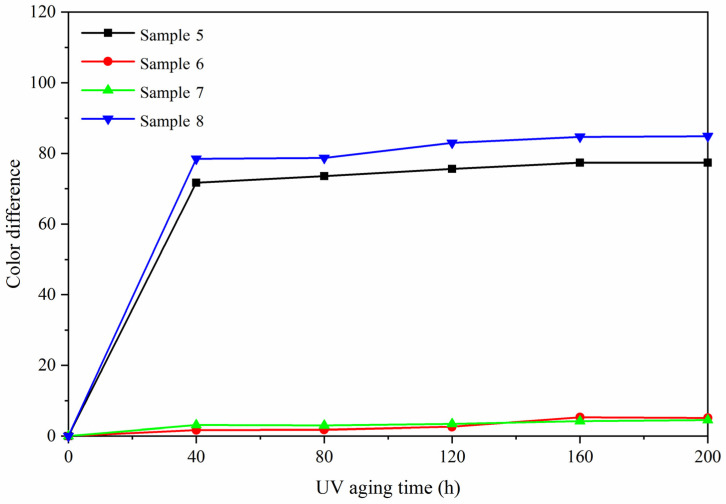
Variation trend of color difference with aging time under UV.

The aging resistance of sample 5 was studied by comparing with the blank samples 6–8. Figure 10, Figure 11 and Figure 12 are trend graphs of the color difference of paint film changing with aging time. After aging at 120 °C (Figure 10), the chromatic aberration of the best sample 5 increased to 13.1, blank sample 6 increased to 10.9, blank sample 7 increased to 7.9, and blank sample 8 increased to 25.5. After the paint film was aged at 160 °C (Figure 11), the chromatic aberration of the best sample 5 increased to 56.0, blank sample 6 increased to 32.6, blank sample 7 increased to 30.4, and blank sample 8 increased to 66.2. After UV aging (Figure 12), the chromatic aberration of the best sample 5 increased to 77.4, blank sample 6 increased to 5.1, blank sample 7 increased to 4.5, and blank sample 8 increased to 84.9. The results showed that the color difference of the same paint film increased with the aging time. After aging, the color difference of samples only added with fluorane microcapsules was larger, which indicated that fluorane microcapsules were unstable during aging and could not keep the chromaticity value of samples. After aging at 160 °C and UV, fluorane was destroyed and degraded to a higher degree [33]. The color difference of water-based paint film changed greatly during thermal aging at 160 °C but changed little during UV aging, which indicated that water-based paint film was not resistant to thermal aging. However, after adding lac resin microcapsules and then adding fluorane microcapsules, the aging color difference of the paint film tended to slow down. This is because the paint film could produce microcracks in the aging process, and the repairing agent flowed out of the lac resin microcapsules, which repaired the microcracks and inhibited the surface damage. On the other hand, the increase of color difference may be due to the color change of wood in the aging environment, which made the color difference of paint film become larger [34].

**Figure 13:**
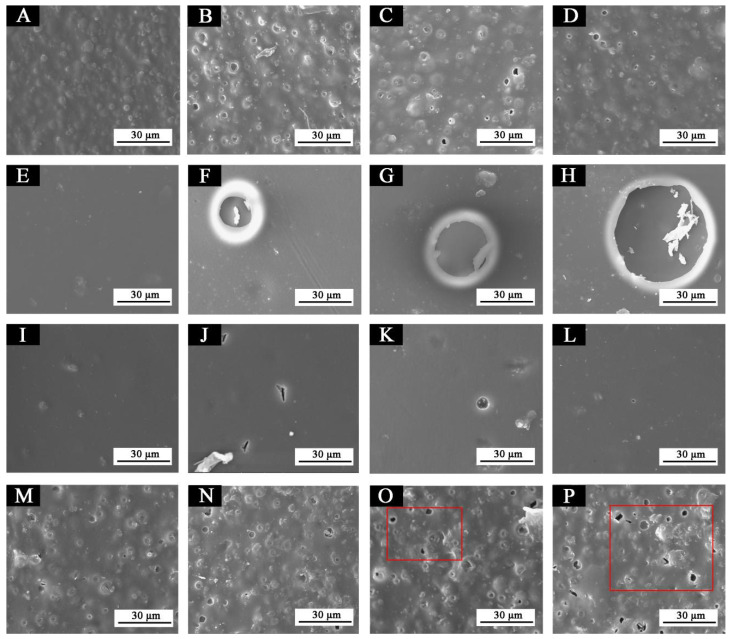
SEM of paint films before and after aging in different environments: (**A**) sample 5, (**B**) sample 5−120 °C, (**C**) sample 5−160 °C, (**D**) sample 5−UV, (**E**) sample 6, (**F**) sample 6−120 °C, (**G**) sample 6−160 °C, (**H**) sample 6−UV, (**I**) sample 7, (**J**) sample 7−120 °C, (**K**) sample 7−160 °C, (**L**) sample 7−UV, (**M**) sample 8, (**N**) sample 8−120 °C, (**O**) sample 8−160 °C, and (**P**) sample 8−UV.

Figure 13 shows SEM images of paint films 5–8 before and after aging in different aging environments. After aging at 120 °C, tiny bubbles began to appear in sample 5. After aging at 160 °C, the number of bubbles increased slightly, and the diameter of bubbles increased slightly, but it was not obvious. After aging in the ultraviolet weathering test chamber, the paint film was in good condition. After aging at 120 °C, larger bubbles began to appear in sample 6 and after aging at 160 °C and in the ultraviolet weathering test chamber, the bubble diameter increased sharply, indicating that the paint film was obviously damaged. After aging at 120 °C and 160 °C, tiny bubbles appeared on the surface of sample 7, but they were not obvious. No obvious bubbles or cracking symptoms appeared after aging in the ultraviolet weathering test chamber, and the paint film was in good condition. After aging at 120 °C, a number of small bubbles began to appear in sample 8 and increased after aging at 160 °C and the ultraviolet weathering test. The results showed that the water-based paint film with fluorane microcapsules or lac resin microcapsules had a certain crack inhibition effect, and the paint film with lac resin microcapsules had a better crack inhibition effect. On the other hand, microcapsules added with lac resin may break when aging, and the repairing agent (lac resin) was released and cross-linked, thus inhibiting the large-scale destruction of the coating microstructure [17,33].

**Figure 14:**
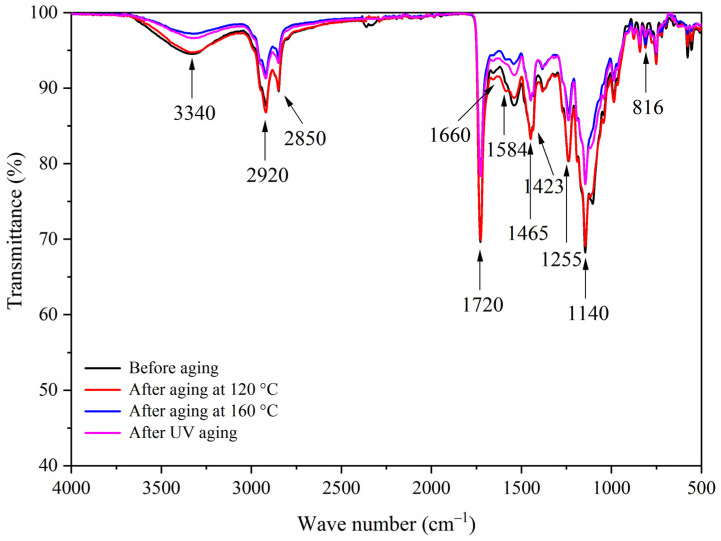
FTIR of sample 5 before and after aging.

**Figure 15:**
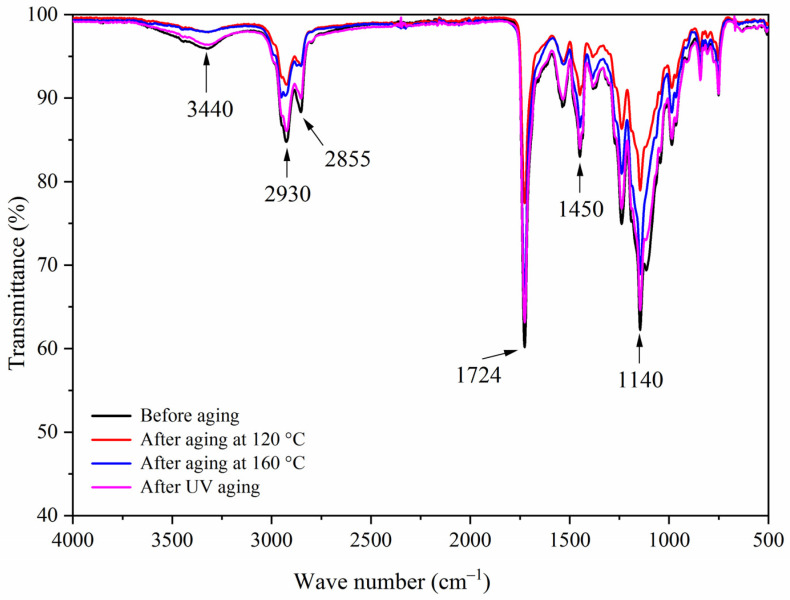
FTIR of sample 6 before and after aging.

**Figure 16:**
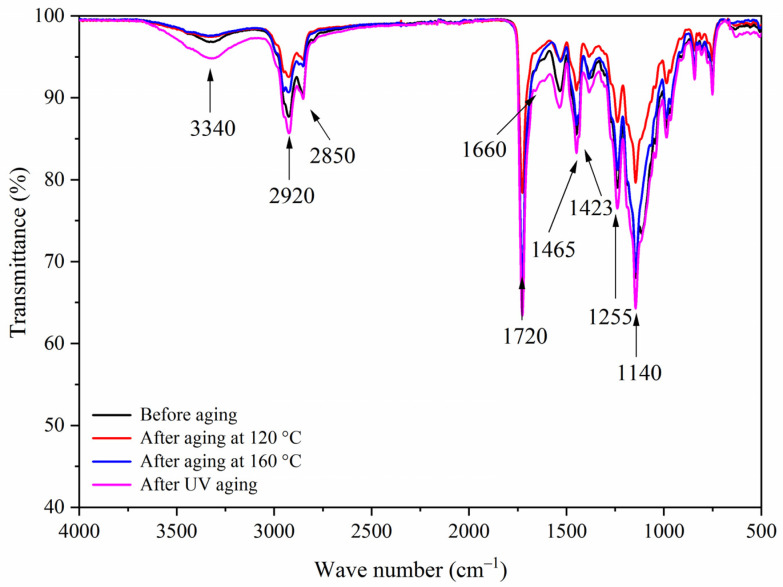
FTIR of sample 7 before and after aging.

**Figure 17:**
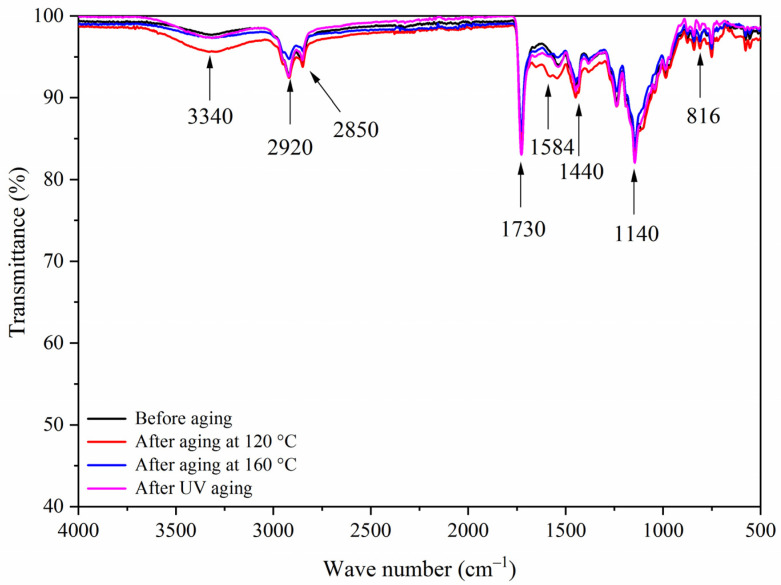
FTIR of sample 8 before and after aging.

Figure 14, Figure 15, Figure 16 and Figure 17 show the infrared spectra of samples 5–8 before and after three aging environments. The stretching vibration absorption of C-O-C in fluorane microcapsules was at 1140 cm^−1^, and the stretching vibration absorption peak and bending vibration absorption peak of the triazine ring were at 1584 cm^−1^ and 816 cm^−1^, respectively. The stretching vibration of the carbonyl group in the conjugated chromogenic structure was near 1725 cm^−1^. The antisymmetric and symmetric stretching vibrations of carbonyl anion COO- in lac resin microcapsules were located at 1465 cm^−1^ and 1423 cm^−1^, respectively, and the stretching vibration of C=O-C in ester molecule was located at 1255 cm^−1^. The 1660 cm^−1^ was the stretching vibration of C=O in urea-formaldehyde resin. The infrared spectrum had the characteristic peaks of fluorane microcapsules and lac resin microcapsules, and the peaks before and after aging did not disappear, which indicated that the aging environment did not cause a chemical reaction of the paint film.

### 3.5. Analysis of Self-Repair and Thermochromic Mechanism

**Figure 18:**
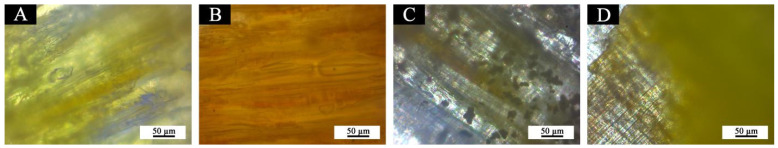
Optical microscope of water-based paint film on the surface of Basswood: (**A**) sample 5, (**B**) sample 6, (**C**) sample 7, (**D**) sample 8.

Figure 18A–D show the optical microscope of samples 5–8. It can be seen from the figure that the pits on the tube wall of the Basswood were arranged in parallel [35]. It can be seen from Figure 18A,D that the water-based paint film of fluorane microcapsules on the surface of Basswood was well dispersed without agglomeration, which may be due to its small particle size of 2.0–4.0 µm. It can be seen from Figure 18A,C that some lac resin microcapsules form agglomeration in the paint film, and the particles were obvious.

**Figure 19:**
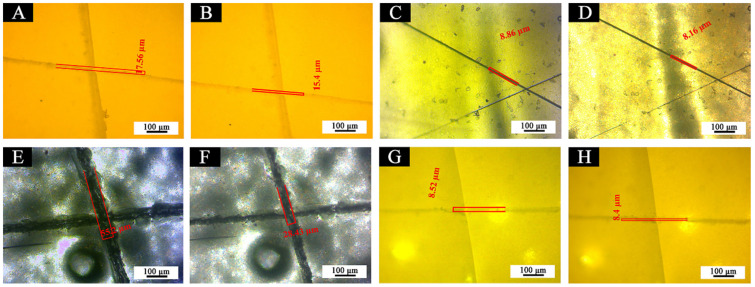
OM of paint film: before self-repairing, (**A**) sample 5, (**C**) sample 6, (**E**) sample 7, and (**G**) sample 8; after scheme (**B**) sample 5, (**D**) sample 6, (**F**) sample 7, and (**H**) sample 8.

Figure 19 is a comparison diagram of crack widths before and after repair of different paint films. Figure 19A was the crack width observed immediately after sample 5 was scratched by a blade, and Figure 19B was the crack width after 5 d. Figure 19C,D show sample 6, Figure 19E,F show sample 7, and Figure 19G,H show sample 8. It can be seen from Table 8 that after the paint film with lac resin microcapsules was left for 5 d, the cracks of the paint film were reduced. The crack width of the best sample 5 before repair was 17.5 µm, and the crack width after 5 d was 15.4 µm, and the gap width of the paint film was reduced by 2.1 µm. The crack width before 7’s repair was 55.2 µm, and after 5 d, the crack width was 28.4 µm, and the gap width was reduced by 26.8 µm. The gap width of the paint film before and after the repair of the paint film without lac resin microcapsules was between 0 and 0.7 µm, which was hardly reduced. Therefore, sample 5 with “10.0% fluorane microcapsule added to the topcoat and 5.0% lac resin microcapsule added to the primer” and sample 7 with “0% fluorane microcapsule added to the topcoat and 15.0% lac resin microcapsule added to the primer” can realize self-repair. The curing of lac coatings depends on the physical volatilization of solvent (ethanol), and there was no chemical reaction in the drying process. When the ethanol volatilizes, lac solidifies into a paint film to block the cracks, so as to repair the cracks. When the paint film with lac resin microcapsules produced microcracks, the microcapsule wall material was broken, the core material lac resin was released, and the microcracks were repaired by crosslinking and curing at room temperature [13].

**Figure 20:**
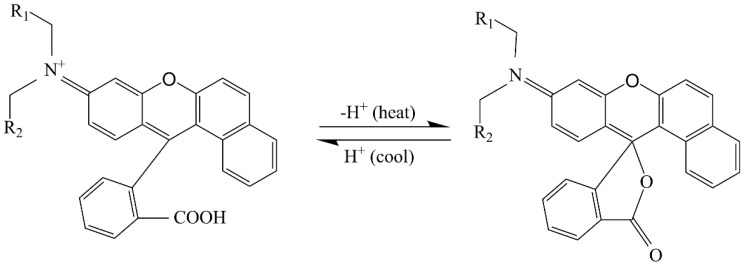
Discoloration principle of fluorane microcapsules.

The color difference comparison of samples 5–8 at 32 °C is shown in Table 8. The color difference of samples 5 and 8 paint films with fluorane microcapsules changed obviously. The color difference of the paint film without fluorane microcapsules had no change and no discoloration effect. The thermochromic principle of fluorane microcapsules is shown in Figure 20. Fluorane thermosensitive dyes were colorless or had only a slight color (proton acceptor), and the central carbon atom and surrounding atoms were bonded to each other through the sp^3^ hybridization orbit to obtain a colorless lactone structure. When the dye came into contact with the chromogenic reagent (proton donor), the lactone ring was opened, the sp^3^ hybrid orbit of the central carbon atom was transformed into an sp^2^ hybrid orbit, and the dye molecule formed a planar conjugated system, which increased the electronic overlapping efficiency and reduced the molecular excitation energy, so that its maximum absorbance shifted to the visible light region, that is, color developed. The lactone ring of these compounds was open at low temperatures and could stay open at low temperatures under the action of a solvent, while the lactone ring was closed and reacted at high temperatures. The effect of temperature made fluorane compounds have reversible thermochromic properties [22].

## 4. Conclusions

An orthogonal experiment showed that the way of adding fluorane microcapsules was the primary factor affecting the color difference of paint film, and the color change temperature of the paint film was 32 °C. The results showed that the paint film with 10.0% fluorane microcapsules in the topcoat and 5.0% lac resin microcapsules in the primer had better comprehensive properties. At this time, the color difference at 32 °C was 68.9, the hardness was 3H, the adhesion grade was 0, the impact resistance was 13.0 kg·cm, the elongation at break was 20.0%, the resistance to red ink was grade 3 with slight marks, and the self-repairing rate was 12.3%. The lac resin microcapsules can decrease the aging color difference of the paint film with fluorane microcapsules and increase the anti-aging property. The self-repair mechanism of lac resin microcapsules is such that when the lac microcapsules are destroyed, the core of the lac coatings flows out, and the ethanol solvent in the lac coating volatilizes physically, which can repair cracks to a certain extent after curing. The thermochromic mechanism of fluorane microcapsules is such that the central carbon atom and surrounding atoms of fluorane combine with each other through the SP^3^ hybrid orbital to obtain a colorless lactone structure. The lactone ring opens and develops color at low temperatures, and the lactone ring closes and does not develop color at high temperatures. This provides a reference for color-changing and self-repairing dual function wood coatings.

## Figures and Tables

**Table 1:** List of experimental materials.

Experimental Materials	Molecular Mass (g/mol)	CAS	Manufacturer
Lac	-	-	Yunnan Yongde Songhua Forest Chemical Products Co., Ltd., Lincang, China
Urea	60.06	57-13-6	Nanjing Quanlong Biotechnology Co., Ltd., Nanjing, China
38.0 % formaldehyde	30.03	50-00-0	
Sodium dodecyl benzene sulfonate (SDBS)	348.48	25155-30-0	
Triethanolamine	149.18	102-71-6	
Citric acid monohydrate	210.14	5949-29-1	
Anhydrous ethanol	46.07	64-17-5	Tianjin Fuyu Fine Chemical Co., Ltd., Tianjin, China
Basswood	-	-	Shanghai Jiangda economic and Trade Co., Ltd., Shanghai, China
Thermochromic microcapsules	-	-	Jinhua Lijin Technology Co., Ltd., Jinhua, China
Dulux water-based primer			Shanghai Keyuan Industrial Co., Ltd., Shanghai, China
Dulux water-based topcoat			

**Table 2:** Experimental design of thermochromic self-repairing paint film.

Sample Number	Content of Fluorane Microcapsule (%)	Content of Lac Resin Microcapsule (%)	Method of Adding Microcapsules
1	10.0	5.0	Fluorane microcapsules were added to the primer, and lac resin microcapsules were added to the topcoat
2	10.0	15.0	Lac resin microcapsules were added to the primer, and fluorane microcapsules were added to the topcoat
3	20.0	5.0	Lac resin microcapsules were added to the primer, and fluorane microcapsules were added to the topcoat
4	20.0	15.0	Fluorane microcapsules were added to the primer, and lac resin microcapsules were added to the topcoat
5	10.0	5.0	Fluorane microcapsules were added to the topcoat, and lac resin microcapsules were added to the primer
6	0	0	
7	0	5.0	
8	10.0	0	

**Table 3:** Ingredients of thermochromic and self-repairing paint film.

Sample Number	Fluorane Microcapsule (%)	Lac Resin Microcapsule (%)	Fluorane Microcapsule (g)	Lac Resin Microcapsule (g)	Primer (g)	Topcoat (g)
1	10.0	5.0	0.2	0.1	1.8	1.9
2	10.0	15.0	0.2	0.3	1.7	1.8
3	20.0	5.0	0.4	0.1	1.9	1.6
4	20.0	15.0	0.4	0.3	1.6	1.7
5	10.0	5.0	0.2	0.1	1.9	1.8
6	0	0	0	0	2.0	2.0
7	0	5.0	0	0.1	2.0	1.9
8	10.0	0	0.2	0	1.8	2.0

**Table 4:** List of experimental instruments.

Instruments	Manufacturer
Heating plate and hand-held thermometer	Dongguan Chang’an Jinfeng electronic tool factory, Dongguan, China
SEGT-J portable colorimeter	Kunshan Lugong Precision Instrument Co., Ltd., Kunshan, China
QHQ portable pencil hardness tester	Dongguan quick measuring instrument Co., Ltd., Dongguan, China
HGQ film scratch tester	
QCJ-50 coating impact tester	Kunshan Haida Precision Instrument Co., Ltd., Kunshan, China
HY-0580 elongation at break tester	Shanghai Hengyi Precision Instrument Co., Ltd., Shanghai, China
Axio Scope A1 optical microscope	Shenzhen Chenqixi Trading Co., Ltd., Shenzhen, China
KYKY-EM6900 scanning electron microscope	Shenzhen Sanhao Instrument Equipment Co., Ltd., Shenzhen, China
XLPE Fourier transform infrared spectrometer	Guangzhou Huruiming Instrument Co., Ltd., Guangzhou, China
202-00B oven	Dongguan Yongxin Electronic Technology Co., Ltd., Dongguan, China
TXH-T ultraviolet weather resistance test oven	

**Table 5:** Orthogonal experimental results of paint film.

Sample Number	Fluorane Microcapsule Content (%)	Lac Resin Microcapsule Content (%)	Method of Adding Microcapsules	Color Difference
1	10.0	5.0	Fluorane microcapsules were added to the primer, and lac resin microcapsules were added to the topcoat	53.2
2	10.0	15.0	Fluorane microcapsules were added to the topcoat, and lac resin microcapsules were added to the primer	79.9
3	20.0	5.0	Fluorane microcapsules were added to the topcoat, and lac resin microcapsules were added to the primer	73.1
4	20.0	15.0	Fluorane microcapsules were added to the primer, and lac resin microcapsules were added to the topcoat	34.1
Mean 1	66.5	63.1	43.6	
Mean 2	53.6	57.0	76.5	
Range	12.9	6.1	32.9	

**Table 6:** Effect of adding methods of fluorane microcapsules on mechanical properties.

Sample Number	Fluorane Microcapsule Addition Method	Impact Resistance (kg·cm)	Hardness (H)	Elongation at Break (%)	Adhesion (Grade)
1	Primer addition	14.0	3H	10.0	0
5	Topcoat addition	13.0	3H	20.0	0

**Table 7:** Effect of methods of adding fluorane microcapsule on liquid resistance.

Sample	Fluorane Microcapsule Addition Method	Red Ink	NaCl	Ethanol	Detergent
1	Primer addition	3	2	1	2
5	Topcoat addition	3	2	2	2

**Table 8:** Comparison of repair rate and color difference.

Sample	Gap Width of Scratch before Repair (µm)	Gap Width of Scratch after Repair (µm)	Gap Width Difference of Scratches before and after Repair (µm)	Repair Rate (%)	Color Difference (32 °C)
5	17.5	15.4	2.1	12.3	68.9
6	8.8	8.1	0.7	7.9	1.8
7	55.2	28.4	26.7	48.4	1.3
8	8.5	8.4	0.1	1.4	75.1

## Data Availability

Not applicable.
